# Use of An Ophthalmology Tutorial to Improve Resident Comfort with the Emergency Eye Exam

**DOI:** 10.21980/J86H0M

**Published:** 2022-10-15

**Authors:** Jessica Pelletier, John Facciani, Francesca Gines, Damon Kuehl

**Affiliations:** * Virginia Tech Carilion School of Medicine, Carilion Clinic, Department of Emergency Medicine, Roanoke, VA; ^ Vistar Eye Center Vision and Surgery Specialists, Roanoke, USA

## Abstract

**Audience:**

This tutorial should be utilized for emergency medicine (EM) interns and junior residents.

**Introduction:**

Ophthalmology is characteristically a weak area in both medical school and resident education. Medical students are rarely given formal didactic education on the use of the slit lamp or a systematic approach to examining the eye. For EM residents, this leads to inefficient and uncomfortable encounters with patients with eye complaints. We sought to develop a comprehensive emergency ophthalmology tutorial utilizing asynchronous learning followed by a hands-on skill session that would address this need.

**Educational Objectives:**

By the end of this small group didactic, learners will be able to: 1) demonstrate ability to focus on the various components of the slit lamp exam 2) demonstrate understanding of a systematic approach to the eye exam 3) demonstrate appropriate use of the Diaton, iCare, and Tonopen tonometers.

**Educational Methods:**

This two-hour small group didactic combines hands-on learning sessions to learn the slit lamp exam and tonometry measurement, with a systematic review of the eye exam to help learners better organize their exams and understand the use of necessary tools.

**Research Methods:**

The emergency ophthalmology tutorial was initially designed as an education project in which we collected pre- and post-participation surveys regarding resident comfort with various components of the emergency eye exam. After the course residents received a post-course survey to complete. Given the positive feedback we received from our residents regarding the tutorial, we applied for Institutional Review Board (IRB) approval to publish our retrospective survey data. Our IRB waived the need for participant consent.

**Results:**

Twelve emergency medicine residents including 11 interns and one post-graduate year (PGY) 2 resident participated in the emergency ophthalmology tutorial as part of our intern boot camp in July of 2021. Twelve PGY-1 residents initially signed up for the course and filled out the pre-participation survey but one of them was not able to attend their scheduled class, so a PGY-2 resident requested to attend.

Prior to the course, we used a Likert scale from 1–7, finding that 61.5% (8/13) of participants felt very uncomfortable with performing slit lamp exams, 84.6% (11/13) felt very uncomfortable with using the Diaton tonometer, 76.9% (10/13) felt very uncomfortable with using the iCare tonometer, and 69.3% (9/13) felt uncomfortable or very uncomfortable with using a systematic approach to examining the eye. After the course, 75% (9/12) of participants felt that the course exceeded expectations in ensuring their ability to perform the subcomponents of the slit lamp exam, 75% (9/12) and 83.3% (10/12) of participants felt that the course exceeded expectations in ensuring their ability to use the Diaton and iCare tonometers, respectively, and 91.7% (11/12) felt that the course exceeded expectations in ensuring their ability to perform a systematic eye exam.

**Discussion:**

Participation in a 2-hour emergency ophthalmology tutorial with assigned asynchronous pre-course work improved emergency medicine resident comfort with various components of the eye exam.

**Topics:**

Emergency ophthalmology, eye exam, slit lamp, tonometry.

## USER GUIDE

**Table:** 

**List of Resources:**	
Abstract	1
User Guide	3
Small Groups Learning Materials	7
Appendix A: Pre-Course Survey	7
Appendix B: Small Group Teaching Materials	8
Appendix C: Post-Course Survey	13
Appendix D: Post-Course Materials	14

**Learner Audience:**

Interns, Junior Residents

**Time Required for Implementation:**

Instructor preparation: 30 minutes

Resident pre-course work: 60 minutes

Course time: 120 minutes (please see comments under

“Recommended Learner: Instructor Ratio” for further details)

**Recommended Number of Learners per Instructor**: 4

Please note that our course was taught in an asynchronous fashion with sessions performed for groups of two residents at a time (2:1 learner: instructor ratio). While this provides learners with more hands-on time with the slit lamp and attention from the instructor, this method is time-intensive on the part of educators. A modified version for small groups with larger learner: instructor ratios could be conducted in stations with one instructor per station.

Residents should be divided into the following stations:

- Station 1 - slit lamp. Half of the residents should start at this station. They should be given 60 minutes at this station to provide adequate time for each of them to practice turning the slit lamp on and off, utilizing and adjusting all necessary buttons and knobs, adjusting the device for their particular patient, and conducting all steps of the slit lamp exam (please see Objectives below). We suggest that the residents rotate through conducting the various steps sequentially (for example, Resident 1 examines the conjunctiva of Resident 2; Resident 2 examines the conjunctiva of Resident 3; etc.) to ensure comfort with each step before moving onto the next step.
- Station 2 - introduction to eye room and systematic approach to the eye exam (see Fig. 2) - 30 minutes, then switch to Station 3.
- Station 3 - tonometry - 30 minutes, then switch to Station 2.

After 60 minutes, residents from Stations 2 and 3 will move to Station 1. Residents from Station 1 will be divided into Stations 2 and 3. After 30 minutes, Stations 2 and 3 will rotate.

**Topics:**

Emergency ophthalmology, eye exam, slit lamp, tonometry.

**Objectives:**

By the end of this small group didactic, learners will be able to:

1. Demonstrate ability to focus on the various components of the slit lamp exam
  - Lids and lashes
  - Conjunctiva and sclera
  - Cornea
  - Anterior chamber
  - Iris
2. Demonstrate understanding of a systematic approach to the eye exam (Fig. 2)
3. Demonstrate appropriate use of the Diaton, iCare, and Tonopen tonometers

### Linked objectives and methods

This tutorial emphasizes the use of hands-on learning as well as immediate and direct feedback to help build foundational emergency medicine skills. The use of pre-course videos and post-course material (see Key Ophthalmology Resources for the Ed Resident) takes advantage of recall to improve chances of solidifying long-term knowledge retention.

First, learners complete pre-learning which reviews slit lamp utilization and common ophthalmologic emergencies by area of the eye visualizable with a slit lamp (Objectives 1 and 2). They then work with an instructor in small groups reviewing how to utilize the slit lamp and demonstrating competence (Objective 1). Next, learners review a systematic approach to the eye exam that they can utilize to examine patients with eye complaints and to organize their presentation of findings to ophthalmology consultants (Objective 2). Finally, participants learn how to use various tonometry measuring devices with an instructor and then demonstrate competence (Objective 3).

### Recommended pre-reading for facilitator

1. *The Wills Eye Manual: Office and Emergency Room Diagnosis and Treatment of Eye Disease.*^1^ The facilitator may want to have it available as a reference during the session.
2. EM:RAP C3 - Eye Trauma.^2^ May read “Summary” or listen to podcast: https://www.emrap.org/c3/playlist/head-andneck/episode/c3eyetrauma/c3eyetrauma
3. EM:RAP C3 - Painful Red Eye.^3^ May read “Summary” or listen to podcast: https://www.emrap.org/c3/playlist/head-andneck/episode/c3painfulredeye/c3painfulredeye
4. EM:RAP C3 - Vision Loss.^4^ May read “Summary” or listen to podcast: https://www.emrap.org/c3/playlist/head-andneck/episode/c3visionloss/c3visionloss

### Learner responsible content (LRC)

Prior to attending the course residents should review a video highlighting the components of the slit lamp exam as well as a slide deck discussing ophthalmologic emergencies by area of the eye that one can visualize with the slit lamp.^6^,^7^

- https://www.youtube.com/watch?v=w9wMJ6job_0&t=8s
- https://aci.health.nsw.gov.au/__data/assets/pdf_file/0010/154963/eem_education_session2.pdf

Residents should complete a pre-course survey (see Appendix A) prior to attending the 2-hour, hands-on slit lamp tutorial.

### Required Materials

Instructors of this course will need ready access to:

- Cotton swabs (to practice inverting eyelids to look for foreign bodies)
- Examination gloves
- Hand sanitizer
- Mobile device with ready access to the internet (to look up images of relevant pathology during each stage of the slit lamp exam)
- Printed copies of the systematic approach to the eye for each participant (Fig. 2)
- Proparacaine and fluorescein (request from pharmacy)
- Slit lamp
- Tonometers, including Diaton, iCare, and Tonopen devices
- Wipes to clean the slit lamp between users

### Results and tips for successful implementation

We collected pre- and post-course survey data to determine whether our emergency ophthalmology tutorial improved resident comfort with the eye exam. Thirteen residents completed pre-course surveys, 12 residents participated in the course, and 12 residents completed post-course surveys. Prior to the course 61.5% (8/13) of participants felt very uncomfortable with performing slit lamp exams, 84.6% (11/13) felt very uncomfortable with using the Diaton tonometer, 76.9% (10/13) felt very uncomfortable with using the iCare tonometer, and 69.3% (9/13) felt uncomfortable or very uncomfortable with using a systematic approach to examining the eye.

The post-course survey prompted the reader to rate whether the tutorial “Does not meet expectations,” “Meets expectations,” or “Exceeds expectations” with regard to each pre-specified course objective. After participation in the course, 75% (9/12) of participants felt that the course exceeded expectations in ensuring their ability to perform the subcomponents of the slit lamp exam, 75% (9/12) and 83.3% (10/12) of participants felt that the course exceeded expectations in ensuring their ability to use the Diaton and iCare tonometers, respectively, and 91.7% (11/12) felt that the course exceeded expectations in ensuring their ability to perform a systematic eye exam.

Since most residents had already been exposed to the Tonopen in medical school (and most of them had used this in the past), data was not collected regarding their level of comfort using the device at the end of the course compared to the outset. Most of them reported significant baseline comfort using the Tonopen device. This did seem a bit incongruous, given their discomfort with the other aspects of the eye exam. Ideally, we would have collected data on their post-course level of comfort to determine whether it had changed.[Fig f1-jetem-7-4-sg1][Fig f2-jetem-7-4-sg1][Table t1-jetem-7-4-sg1]

Here we describe our methods and the effectiveness based on before-and-after survey data of an emergency ophthalmology tutorial to improve resident comfort with performance of slit lamp exams, use of tonometry, and a systematic approach to the eye exam. The majority of residents felt uncomfortable or very uncomfortable with these various components of the emergency eye exam prior to participation in the course.

The course had the following objectives:

1. Demonstrate ability to focus on the various components of the slit lamp exam
  - Lids and lashes
  - Conjunctiva and sclera
  - Cornea
  - Anterior chamber
  - Iris
2. Demonstrate understanding of a systematic approach to the eye exam (Fig. 2)
3. Demonstrate appropriate use of the Diaton, iCare, and Tonopen tonometers

The majority of residents rated the tutorial as exceeding expectations in meeting its objectives, suggesting that resident comfort with performance of slit lamp exams, tonometry, and using a systematic approach to the examination of the eye improved via participation in this course.

Our goal was to evaluate baseline comfort with performance of the aforementioned procedures and then evaluate whether residents felt that they benefited from the course. A comment should be made on the limitations of our data. Survey-based research is a low level of evidence for the effectiveness of an intervention. Ideally, to determine whether our course was beneficial in improving resident comfort with the emergency ophthalmology examination, we would utilize a blinded evaluator to prospectively evaluate residents who participated in the course vs. those who did not participate. A standardized evaluation tool such as the General Approach to Procedures component of the ACGME Emergency Medicine Milestones 2.0 could be utilized to evaluate resident ophthalmology examinations. We hope that as we and others disseminate curricula for improving emergency ophthalmology education, and as tools emerge for evaluating resident competency in this area, we can ensure that our emergency medicine residents effectively evaluate and treat patients with ophthalmologic complaints.

Another limitation is that the course was taught by a single medical educator. It is unclear whether the results would be reproducible if the course were taught by a provider with more or less experience utilizing the examination techniques reviewed in the course.

Lastly, residents practiced slit lamp examinations on one another, and few of them had abnormal findings on slit lamp examination. In order to create a more robust educational experience, volunteer patients with abnormal eye examinations (perhaps from an ophthalmology clinic) could be asked to serve as models for Station 1 of the small group session. This would require more time and planning but would be worth considering if you plan to implement and build on our curriculum at your institution.

One of our course objectives included demonstrating the ability to perform tonometry using the Tonopen. We collected baseline pre-course survey data but neglected to collect post-course survey data regarding whether this objective was met.

One of the participating residents was a PGY-2 resident and may have had significantly more experience with the techniques reviewed in the course than their PGY-1 peers. This may have affected the overall survey data results, skewing the baseline data toward higher levels of comfort than might have been seen otherwise.

In summary, we describe the use of a hands-on emergency ophthalmology tutorial with associated asynchronous pre-course work designed to improve subjective, self-reported resident comfort with the performance of the emergency eye exam. Our retrospective survey data demonstrate improved subjective, self-reported resident comfort with the various components of the exam covered in the course, including the slit lamp exam, tonometry using the iCare and Diaton tonometers, and examining the eye using a systematic approach. Ophthalmology education prior to residency is limited, and most residents have little exposure to ophthalmology education.^5^ Our hope in publishing our survey data and course materials is that other emergency medicine residency programs may be able to adopt our curricula to improve the subjective, self-reported comfort of their residents with performing emergency eye exams.

## Figures and Tables

**Figure 3 f1-jetem-7-4-sg1:**
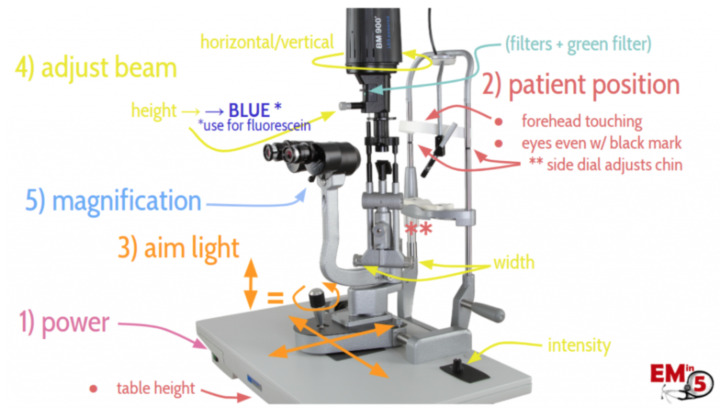
Pre-course survey data demonstrating baseline resident comfort with performance of a slit lamp exam, use of the Diaton tonometer, use of the iCare tonometer, and using a systematic approach to examining the eye. Degree of comfort was assessed on a scale from 1 to 5 with 1 being “very uncomfortable” and 5 being “very comfortable.”

**Figure 4 f2-jetem-7-4-sg1:**
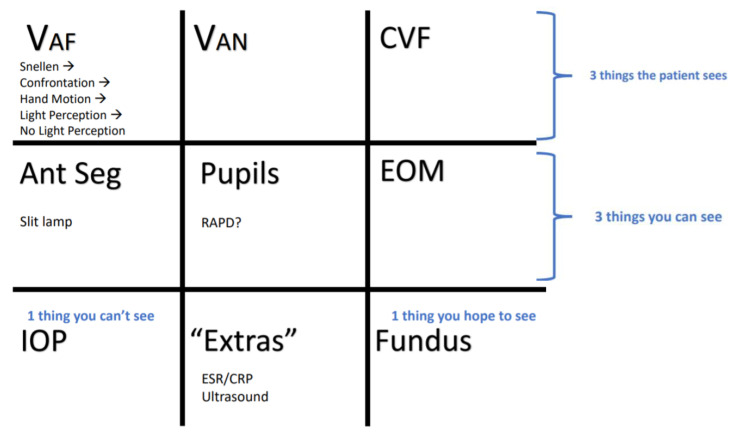
Post-course survey data demonstrating resident comfort with performance of a slit lamp exam, use of the Diaton tonometer, use of the iCare tonometer, and using a systematic approach to examining the eye.

**Table 1 t1-jetem-7-4-sg1:** Resident feedback to the question, “Do you have any other suggestions to make this tutorial better?”

Resident 1	*Great overview and tutorial. Thank you!*
Resident 2	*Such an amazing introduction. I greatly appreciate the care and time put into this. Serving yourself up as the model for tonometry was 5 steps beyond anything I would have expected. Thank you.*
Resident 3	*Honestly the most comprehensive and clinical high yield discussion on optho [*sic*] I have had to date! Thank you for putting this together it was invaluable to my optho knowledge in the ED.*
