# Supplementary material for: Use of An Ophthalmology Tutorial to Improve Resident Comfort with the Emergency Eye Exam
Source: J Educ Teach Emerg Med. 2022 Oct 15;7(4):SG1–SG14. doi: 10.21980/J86H0M (PMC10332671; doi:10.21980/J86H0M)
Supplement: Supplementary file 1 [file JETem-7-4-SG1-AppendixA.docx]

Appendix A:

Ophthalmology Tutorial Pre-Course Survey

Residents should complete a pre-course survey prior to attending the 2-hour, hands-on slit lamp tutorial. Google forms can be used.

**Pre-Course Survey:**

| How comfortable do you feel with using a slit lamp at this time? | | | | |
| --- | --- | --- | --- | --- |
| 1-Very UNcomfortable | 2 | 3-Neutral | 4 | 5-Very Comfortable |
| How comfortable do you feel with using a systematic approach to examining an eye at this time? | | | | |
| 1-Very UNcomfortable | 2 | 3-Neutral | 4 | 5-Very Comfortable |
| How comfortable do you feel with using the Diaton tonometer? | | | | |
| 1-Very UNcomfortable | 2 | 3-Neutral | 4 | 5-Very Comfortable |
| How comfortable do you feel with using the iCare tonometer? | | | | |
| 1-Very UNcomfortable | 2 | 3-Neutral | 4 | 5-Very Comfortable |
