# Supplementary material for: Use of An Ophthalmology Tutorial to Improve Resident Comfort with the Emergency Eye Exam
Source: J Educ Teach Emerg Med. 2022 Oct 15;7(4):SG1–SG14. doi: 10.21980/J86H0M (PMC10332671; doi:10.21980/J86H0M)
Supplement: Supplementary file 2 [file JETem-7-4-SG1-AppendixB.docx]

Appendix B:

Small Group Application Exercise (sGAE)

**Required Materials:**

Instructors of this course will need ready access to:

- Cotton swabs (to practice inverting eyelids to look for foreign bodies)
- Examination gloves
- Hand sanitizer
- Mobile device with ready access to the internet (to look up images of relevant pathology during each stage of the slit lamp exam)
- Printed copies of the systematic approach to the eye for each participant (Fig. 2)
- Proparacaine and fluorescein (request from pharmacy)
- Slit lamp
- Tonometers, including Diaton, iCare, and Tonopen devices
- Wipes to clean the slit lamp between users

**Stations:**

STATION 1 - SLIT LAMP

STATION 2 - INTRODUCTION TO EYE ROOM AND SYSTEMATIC APPROACH TO THE EYE EXAM

STATION 3 - TONOMETRY

STATION 1 - SLIT LAMP

Station 1 involves an introduction to the use of the slit lamp.[^8^](https://www.zotero.org/google-docs/?kvU1rI) Residents will trade off going through the following steps, addressing Objective 1:

1. Turn on the slit lamp. Our institution’s slit lamp has both a wall timer and a power switch for the device, and both must be turned on in order to use the device. Ensure that you clarify the unique aspects of turning on your institution’s device.
2. Adjust the eyepieces for the width and magnification necessary for the user’s own eyes.
3. Unlock the base of the slit lamp by loosening the screw at the bottom right of the base of the device.
4. Aim the light. This is accomplished by grasping the joystick with one hand and moving towards the patient to magnify the eye, and away from the patient to make the image smaller. We practiced with the dominant hand first but the residents should be encouraged to practice with both hands during the course for greater ease with examining each eye.
5. Adjust the light beam. Residents should practice changing the color of the light. Discuss that they will almost always use bright white light except when using cobalt blue light for fluorescein staining. They should also practice making the light beam taller vs. shorter, and making the light beam wider vs. thinner. Residents should learn how to rotate the beam and how to measure the size of lesions on the surface of the eye by adjusting the beam to the size of the lesion and using the scale on the top of the slit lamp.
6. Make the patient comfortable. Residents should practice adjusting the chin rest, forehead strap, and chair for each other to ensure that they can obtain ideal views of the eye.

The steps to using the slit lamp exam above should be performed sequentially. For example, Resident 1 should complete step 1, then Resident 2 should complete step 1. Next, Resident 1 should complete step 1 and step 2, then Resident 2 should complete step 1 and step 2. All participants should proceed through the aforementioned steps in this manner. At this point they should run through the entire sequence once more to ensure that they feel comfortable with turning the slit lamp on and off, adjusting the various controls, and making the patient comfortable.

Residents will next practice examining each area of the eye on one another using the slit lamp. This includes lids and lashes, conjunctiva and sclera, cornea, anterior chamber, and iris. At each step of the slit lamp exam, residents are shown pictures on a mobile phone of what they should be looking for (see <https://aci.health.nsw.gov.au/__data/assets/pdf_file/0010/154963/eem_education_session2.pdf>, part of the pre-course work described above). Each resident should try to identify the given structure (for example, cornea) using the slit lamp, and the course instructor will take a look through the slit lamp to ensure that the resident has positioned the equipment correctly. The residents should be given direct, real-time feedback if they are visualizing the components of the eye exam incorrectly, and they are instructed as to how to make adjustments to the equipment in order to obtain the correct views. They are not permitted to move on to the next step until they feel comfortable visualizing each component of the slit lamp exam.

During the lids and lashes portion of the slit lamp exam, residents should practice inverting each other’s eyelids in the same fashion that they would look for foreign bodies. If their co-residents are willing to undergo this, residents should practice performing a fluorescein stain with both the Woods lamp and the cobalt blue light on the slit lamp.

Throughout the course, the clinical relevance of each component of the examination should be discussed by the instructor. For example, when examining the conjunctiva, the residents are prompted as to what pathology they might be looking for in various patients presenting with foreign body sensation, discharge, redness, or swelling. After the residents feel comfortable visualizing the normal conjunctiva of their colleague through the slit lamp, images of common conjunctival pathology are reviewed on a mobile device (see <https://aci.health.nsw.gov.au/__data/assets/pdf_file/0010/154963/eem_education_session2.pdf>) and appropriate treatment regimens for these disease processes should be discussed. A similar approach should be taken for each remaining component of the slit lamp exam (lids and lashes, sclera, cornea, anterior chamber, and iris).


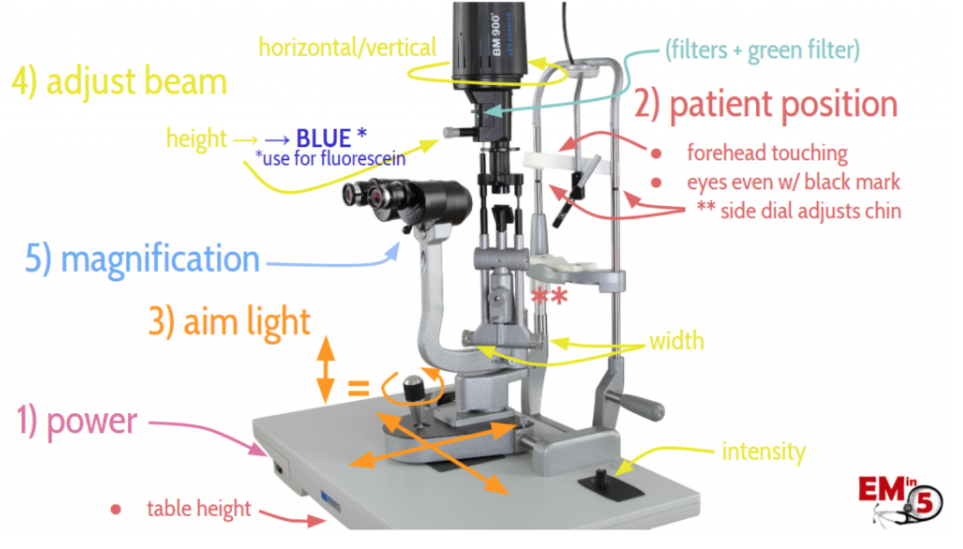


**Figure 1**: Labeled diagram of a standard slit lamp. This still image can be found in the blog post “EMin5: Slit Lamp Anatomy” which includes a video on utilizing the slit lamp and step-wise images highlighting how to use the various buttons and knobs on a standard slit lamp. Residents are provided access to this link after the course to solidify their understanding (see Key Ophthalmology Resources for The Ed Resident).[^8^](https://www.zotero.org/google-docs/?KmjrV5)

Pickens A. Slit Lamp Anatomy. In: EMin5. Accessed September 23, 2022. CC BY 4.0. At:

<http://www.emdocs.net/emin5-slit-lamp-anatomy/7>

STATION 2 - INTRODUCTION TO EYE ROOM AND SYSTEMATIC APPROACH TO THE EYE EXAM

A novel systematic approach to the eye exam (Fig. 2) should be presented and practiced in order to address Objective 2. This tool was developed by one of the authors (an ophthalmologist) with inspiration from a lecture by an ophthalmology colleague (please see “Special Thanks”). *Please note that this tool has not yet been validated externally*. Residents should be instructed to draw the tic-tac-toe diagram themselves each time they examine a patient with an eye complaint and fill in each box with their findings. Residents are told to begin in the top left box and work their way from left to right, then proceed with the second row of boxes, followed by the third row of boxes in the same fashion. The top row is entitled "3 things the patient sees," indicating that the patient will present with visual complaints such as loss of vision if they have abnormal findings in one of these categories. The middle row, or "3 things you can see," refers to physical abnormalities on slit lamp, pupillary, or eye movement testing that will be detected by the examiner. In the bottom row, IOP (intraocular pressure) is labeled "1 thing you can't see" since this requires mechanical testing with tonometry. Residents are encouraged to perform fundoscopic examinations, but with the understanding that we usually do not perform pupillary dilation in the ED, making the ability to visualize the fundus limited (hence, "1 thing you hope to see"). "Extras'' refers to adjunctive testing that may not be necessary for every eye complaint and must be tailored to the patient.


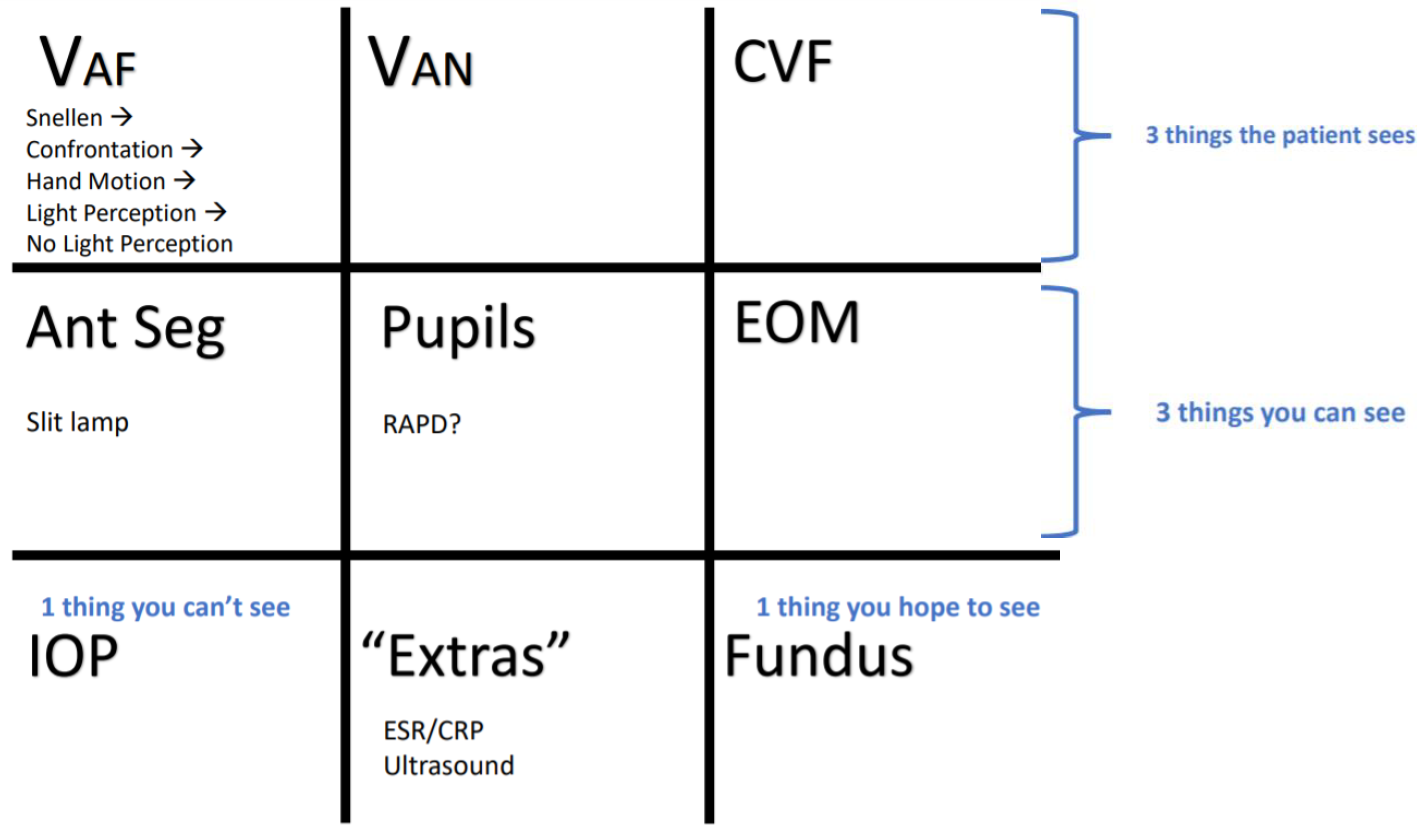


**Figure 2**: Systematic Approach to the Eye Exam. VAF = visual acuity far, VAN = visual acuity near, CVF = confrontational visual fields, Ant Seg = anterior segment, RAPD = relative afferent pupillary defect, EOM = extraocular movements, IOP = intraocular pressure, ESR = erythrocyte sedimentation rate, CRP = C-reactive protein.

STATION 3 - TONOMETRY

Residents in this station will review and practice tonometry in order to address Objective 3. The instructor should provide a demonstration of how to use the Diaton, iCare, and Tonopen tonometry devices using their hand as a model eye. After the demonstration, the residents will practice using each type of tonometry device on their colleagues or the instructor until they feel sufficiently comfortable that they can obtain an intraocular pressure (IOP) without difficulty.
