# Supplementary material for: Use of An Ophthalmology Tutorial to Improve Resident Comfort with the Emergency Eye Exam
Source: J Educ Teach Emerg Med. 2022 Oct 15;7(4):SG1–SG14. doi: 10.21980/J86H0M (PMC10332671; doi:10.21980/J86H0M)
Supplement: Supplementary file 3 [file JETem-7-4-SG1-AppendixC.docx]

Appendix C:

Ophthalmology Tutorial Post-Course Survey

Participants will complete a post-course survey (see below) which should be emailed to them immediately after the tutorial is complete. We used Google forms, and email addresses were not collected to ensure that all survey data was collected anonymously.

**Post-Course Survey:**

Please select whether the course accomplished its intended objectives.

| Demonstrate ability to perform the various components of the slit lamp exam: lids and lashes, conjunctiva and sclera, cornea, anterior chamber, iris | | |
| --- | --- | --- |
| Does Not Meet Expectations | Meets Expectations | Exceeds Expectations |
| Demonstrate understanding of a systematic approach to the eye exam | | |
| Does Not Meet Expectations | Meets Expectations | Exceeds Expectations |
| Demonstrate appropriate use of the iCare tonometer | | |
| Does Not Meet Expectations | Meets Expectations | Exceeds Expectations |
| Demonstrate appropriate use of the Diaton tonometer | | |
| Does Not Meet Expectations | Meets Expectations | Exceeds Expectations |
