# Supplementary material for: Use of An Ophthalmology Tutorial to Improve Resident Comfort with the Emergency Eye Exam
Source: J Educ Teach Emerg Med. 2022 Oct 15;7(4):SG1–SG14. doi: 10.21980/J86H0M (PMC10332671; doi:10.21980/J86H0M)
Supplement: Supplementary file 4 [file JETem-7-4-SG1-AppendixD.docx]

Appendix D:

Post-Course Materials

Participants should be sent the following post-course materials in an email along with their post-course survey in order to solidify their learning:

**KEY OPHTHALMOLOGY RESOURCES FOR THE ED RESIDENT**

- C3 - Painful Red Eye. This will give you a solid foundation regarding the diagnosis, workup, and management of ophthalmologic emergencies.[^3^](https://www.zotero.org/google-docs/?O06mC2)
- EMin5: Slit Lamp Anatomy – quick article that reminds you how to use the Slit Lamp: [http://www.emdocs.net/emin5-slit-lamp-anatomy/7](http://www.emdocs.net/emin5-slit-lamp-anatomy/)[^8^](https://www.zotero.org/google-docs/?jDT0WU)
- Eye Chart app – allows you to randomize the Snellen chart, just in case your patient is memorizing the lines when you test the first eye: <https://apps.apple.com/us/app/eyechart-vision-screening/id293163439>[^9^](https://www.zotero.org/google-docs/?j09lgo)
- Ophthalmic Drops 101 – handy color-coded chart of all the drops you need to know: <https://www.aao.org/young-ophthalmologists/yo-info/article/ophthalmic-drops-101>[^10^](https://www.zotero.org/google-docs/?TjKjuB)
- Tim Root – ophthalmology videos made basic and easy to understand: <https://timroot.com/videos/>[^11^](https://www.zotero.org/google-docs/?B8PfXP)
- The Wills Eye Manual – you can get this for less than $20 online. This is a comprehensive guide to eye pathology and covers more than what we need to know for the ED but can be an excellent reference.[^1^](https://www.zotero.org/google-docs/?XW9NOo)

**SLIT LAMP DEEP DIVES**

These videos are really helpful after you have played with the slit lamp:

- Adel Abdelshafik: <https://www.youtube.com/watch?v=ePnhGyQCXmo>
  - You don’t need to watch the whole thing. Key portions:
    - 6:00-25:43 figuring out what layers things are in with the slit beam
    - 27:16-32:45 flare in the anterior chamber
    - 32:45-34:36 the importance of having your light beam and microscope at an angle
    - 37:21-44:19 retro illumination
    - 56:13-57:44 measuring stuff with the slit beam[^12^](https://www.zotero.org/google-docs/?osu8Qy)
- Tim Root: [https://www.youtube.com/watch?v=w9wMJ6job_0&t=10s55](https://www.youtube.com/watch?v=w9wMJ6job_0&t=10s)
  - Start at 3:20[^6^](https://www.zotero.org/google-docs/?yrNRgm)
- Doctor Eyeball MD: <https://www.youtube.com/watch?v=SQAbhoj82Po&t=899s>
  - Stop at 10:55[^13^](https://www.zotero.org/google-docs/?im92gU)
